# Quantitative Proteomic Analysis Indicates That *Pggt1b* Deficiency Promotes Cytokine Secretion in Resiquimod‐Stimulated Bone Marrow‐Derived Macrophages via the NF‐κB Pathway

**DOI:** 10.1002/iid3.70185

**Published:** 2025-04-07

**Authors:** Shanshan Yu, Xuecui Wei, Fangyuan Long, Heng Gu, Zhimin Hao

**Affiliations:** ^1^ Institute of Dermatology Chinese Academy of Medical Sciences & Peking Union Medical College Nanjing China; ^2^ School of Public Health Nanjing Medical University Nanjing China

**Keywords:** BMDM, LC‐MS/MS, NF‐κB signaling pathway, Pggt1b, psoriasis, R848

## Abstract

**Background:**

Psoriasis is a systemic inflammatory skin disease mediated by the innate and adaptive immune systems. Recent studies have indicated that macrophages may contribute to the pathogenesis of psoriasis. However, the role of macrophage protein geranylgeranyl transferase type‐1β subunit (PGGT1B) in psoriasis is unclear. In this study, we aimed to establish how a reduction in Pggt1b expression in monocytes influences the onset and progression of psoriasis.

**Methods:**

Myeloid cell‐specific Pggt1b knockout mice were generated, and their bone marrow‐derived macrophages (BMDMs) were stimulated with resiquimod (R848) to mimic the psoriatic immune microenvironment. The proteomic analysis enabled us to identify 17 differentially expressed proteins associated with Pggt1b deficiency in the psoriasis macrophage model (folded change ≥ 1.3 and *p* < 0.05). Gene Ontology and Kyoto Encyclopedia of Genes and Genomes enrichment was performed. Quantitative reverse transcription‐polymerase chain reaction (qRT‐PCR) and western blot assays were used to verify the differentially expressed proteins and signaling pathways. Finally, an enzyme‐linked immunosorbent assay was used to verify the expression of the key inflammatory cytokine interleukin (IL)‐1β.

**Results:**

In total, six proteins (Dlgap5, Fas, Fnta, Nlrp3, Cd14, and Ticam2) were identified as hub proteins. Furthermore, we found that Pggt1b might mediate R848‐induced inflammation via the small G‐proteins Rac1 or Cdc42. We found that Pggt1b positively regulates pro‐inflammatory responses in R848‐stimulated BMDMs via the NF‐κB signaling pathway.

**Conclusions:**

This study clarified that PGGT1B affected the synthesis of inflammatory cytokines via NF‐κB pathway and provided insights into the mechanisms underlying immune responses and inflammation.

## Graphical abstract

In Pggt1b‐deficient macrophages, TLR4 may be in a highly activated state and can be activated by R848 or DAMPS, which strongly correlated with increased cytokine production, interferon‐γ production, augmented amide binding and transferase activity, and the activation of NF‐κB and/or Nlrp3 inflammasome pathways.

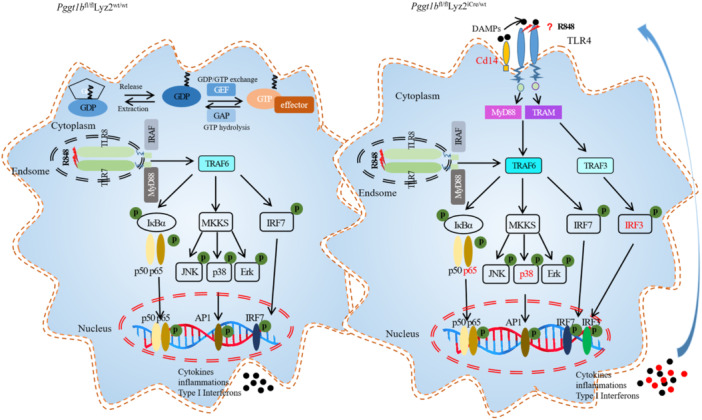

**Abbreviations**
BMDMs: bone marrow‐derived macrophages
BP: biological process
CC: cellular component
DEPs: differentially expressed proteins
GO: Gene Ontology
KEGG: Kyoto Encyclopedia of Genes and Genomes
LC‐MS/MS: liquid chromatography tandem mass spectrometry
NF‐κB: nuclear factor κ‐light‐chain‐enhancer of activated B cells
NLRP3: NOD‐, LRR‐, and pyrin domain‐containing protein 3
PBMC: peripheral blood mononuclear cell
Pggt1b: protein geranylgeranyl transferase type I β subunit
qRT‐PCR: quantitative reverse transcription‐polymerase chain reaction
TLR: Toll‐like receptor
TNF‐α: tumor necrosis factor α

## 1 Introduction

Psoriasis is an immune‐mediated polygenic inflammatory skin disease that affects 2%–3% of the global population [1]. Multiple factors play important roles in the pathogenesis of psoriasis [2, 3]. A large number of studies have confirmed that the occurrence and development of psoriasis involve the abnormal activation of various immune cells and the imbalance of complex inflammatory signal networks. Among them, macrophages play a vital role in the immunopathological process of psoriasis. They promote the cascade amplification of inflammatory reaction by secreting a variety of cytokines, such as tumor necrosis factor α (TNF‐α) and interleukin 1β (IL‐1β), which leads to the excessive proliferation of keratinocytes and the appearance of skin inflammation [4, 5, 6, 7, 8].

In recent years, the in‐depth study of the immune system regulation mechanism reveals the potential role of many new molecules and signaling pathways in the pathogenesis of psoriasis. The protein *O*‐GlcNAc transferase (OGT) and its related molecules have gradually attracted attention in the regulation of the immune system. As an important protein, geranylgeranyl transferase type‐1 β subunit (PGGT1B) plays a key role in the activation of immune cells and inflammatory reactions. Under normal circumstances, the acylation of protein geranyl geranium helps to maintain the normal function of immune cells. However, when PGGT1B is defective, it may affect this modification process, resulting in abnormal immune cell function [9]. Studies have shown that myeloid PGGT1B deficiency will lead to abnormal functions of immune cells (such as macrophages and neutrophils) [10]. When stimulated, these cells will release more inflammatory factors, such as IL‐1β and TNF‐α. At the same time, Chen et al. have found that PGGT1B in peripheral blood mononuclear cells of patients with psoriasis is significantly decreased. At the same time, the lack of PGGT1B may be related to the upregulation of pro‐inflammatory cytokines in psoriasis. It shows that PGGT1B can be used as a potential biomarker for the diagnosis and treatment of psoriasis [9]. Based on these preliminary results, we speculate that PGGT1B may play a potential role in the inflammatory response of psoriasis. However, there is still a great knowledge gap about how it participates in the key pathological process of psoriasis and its specific mechanism in patients with psoriasis.

The activation of the NF‐κB signaling pathway is the key link in the inflammatory reaction of psoriasis, which is involved in regulating the proliferation of keratinocytes, the activation of immune cells, and the production of inflammatory cytokines [11, 12]. Therefore, PGGT1B may regulate the inflammatory response of cells by influencing the activity of NF‐κB (nuclear factor κB) signal molecules. In view of the decreased expression of PGGT1B in patients with psoriasis, this protein is the key regulator of inflammatory cytokine production. We hypothesized that the lack of macrophages in PGGT1B may aggravate the pathogenesis of psoriasis related to inflammatory signals. The purpose of this study is to explore the role of PGGT1B in psoriasis and its potential mechanism. The Toll‐like receptor (TLR)7/8 agonist imiquimod (IMQ) or resiquimod (R848) is commonly used to study psoriatic immune cells [13, 14, 15]. Therefore, we used R848 to simulate the immune microenvironment of psoriasis, stimulate mouse bone marrow‐derived macrophages (BMDMs), and study the influence of decreased expression of monocyte Pggt1b on the pathogenesis and progress of inflammatory diseases, thus being able to provide the pathogenesis of psoriasis.

## 2 Materials and Methods

**Figure 1:**
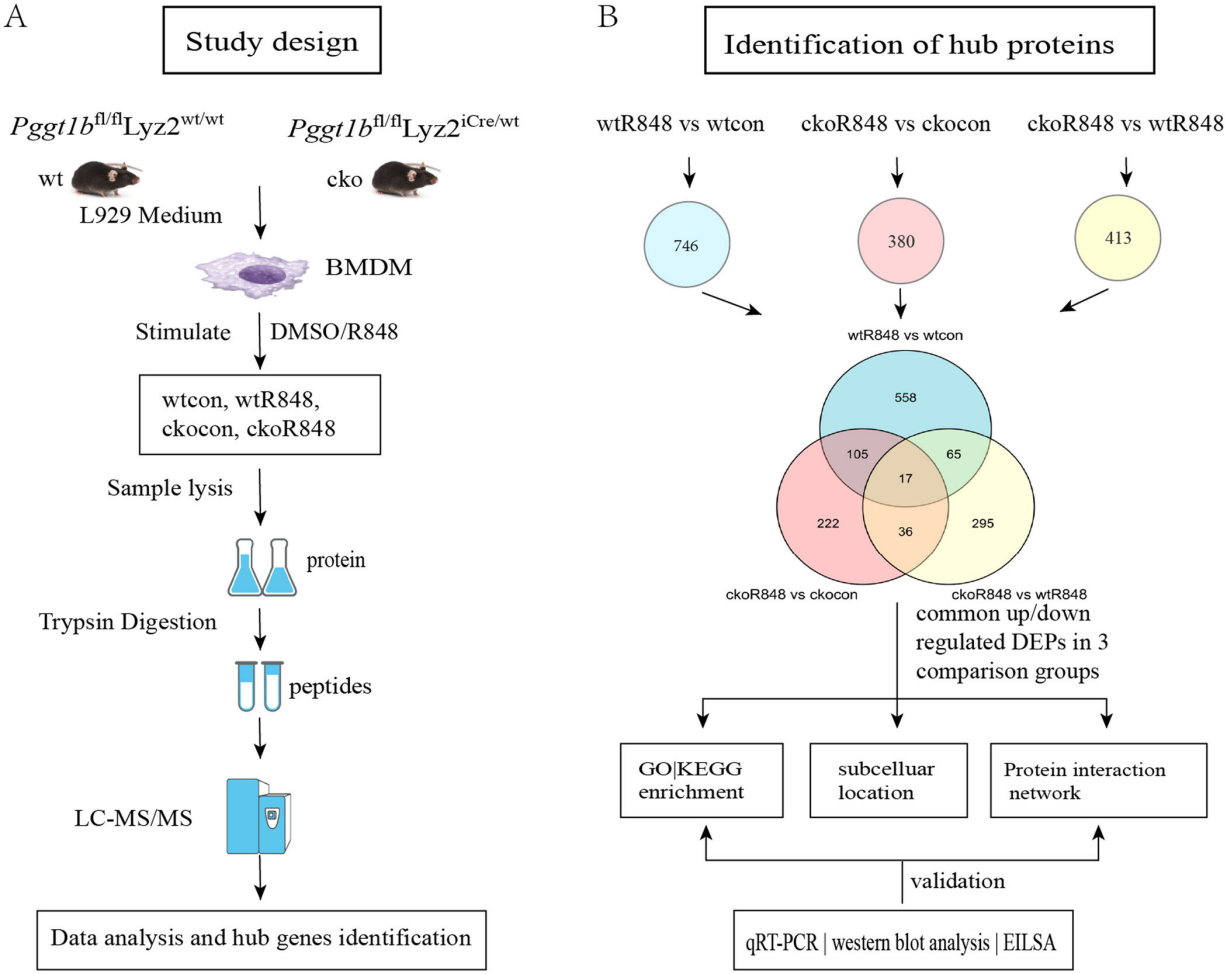
Study flow diagram. (A) Flow diagram of the preparation of bone marrow‐derived macrophage (BMDM) protein populations and the mass spectrometry‐based quantitative proteomics strategy. (B) Flowchart of protein identification.

A flowchart of the study design is shown in Figure 1A, and details of the data analyses and verification processes are presented in Figure 1B.

### 2.1 Mice

*Pggt1b* ^em1Cflox^/Gpt mice were obtained from GemPharmatech (Nanjing, China), from which *Pggt1b* ^fl/fl^Lyz2^iCre/wt^ and littermate control *Pggt1b* ^fl/fl^Lyz2^wt/wt^ mice were generated by initially crossing the *Pggt1b* ^em1Cflox^/Gpt mice with B6/JGpt‐Lyz2^em1Cin(iCre)^/Gpt mice, followed by intercrossing the offspring *Pggt1b* ^fl/fl^Lyz2‐Cre mice. Littermate controls were used in the experiments. For each mouse genotype, BMDMs were extracted from a single mouse of the same sex, and each experiment was repeated three times (twice with a female and once with a male). The mouse strains were maintained in a specific pathogen‐free environment in the Experimental Animal Center, Institute of Dermatology, Chinese Academy of Medical Sciences, and Peking Union Medical College. The animal procedures performed in this study were approved by The Ethics Committee of Dermatology Hospital, Chinese Academy of Medical Sciences (approval no. 22‐DW‐007).

### 2.2 BMDM Culture and Stimulation

The details of BMDM culture and stimulation are described in Supporting Information.

### 2.3 LC‐MS/MS Analysis

The details of LC‐MS/MS analysis are described in Supporting Information.

### 2.4 Bioinformatics

The details of bioinformatics are described in Supporting Information.

### 2.5 Quantitative Real‐Time PCR

The details of qRT‐PCR are described in Supporting Information. The gene‐specific primers used in this study are listed in Table 1.

**Table 1:** Primer sequences used in quantitative reverse transcription‐polymerase chain reaction (qRT‐PCR).

Gene	Forward (5'–3')	Reverse (5'–3')
*Myadm*	ATGCCGGTAACAGTAACTCGT	CCACACAGGTGGATATTAGCTG
*Gstm2*	ACACCCGCATACAGTTGGC	TGCTTGCCCAGAAACTCAGAG
*Gnptg*	AACACATTCGGGCTGAATAACC	CCACTAGGCTAAAGCACTTGC
*Acy1*	CAACCCAATCCAGACTATGGAGG	GTGGGAGTTTAGCAAGATGGAG
*Ephx1*	GGAGACCTTACCACTTGAAGATG	GCCCGGAACCTATCTATCCTCT
*Sil1*	CCTTCAACTAGGATGGCCTCT	CCTCCAGGATCTCGGTGTCT
*Gys1*	GAACGCAGTGCTTTTCGAGG	CCAGATAGTAGTTGTCACCCCAT
*Stfa1*	TTCTTCTCAGTGTCCAAGCCA	ATATTTTCTCCAGCGACGACT
*Fas*	TATCAAGGAGGCCCATTTTGC	TGTTTCCACTTCTAAACCATGCT
*Nlrp3*	ATTACCCGCCCGAGAAAGG	TCGCAGCAAAGATCCACACAG
*Cd14*	CTCTGTCCTTAAAGCGGCTTAC	GTTGCGGAGGTTCAAGATGTT
*Fnta*	CCCTATGGACGACGGGTTTC	TGATCTGGACCACTGGGTTAG
*Siglec1*	CAGGGCATCCTCGACTGTC	GGAGCATCGTGAAGTTGGTTG
*Ticam2*	CGATCAAGACGGCCATGAGTC	CTCGTCGGTGTCATCTTCTGC
*Eogt*	TTAATGCTGCTTGTCTTTGGAGT	GCAACATGCCTGTTATTGTGC
*Dlgap5*	CAGTCCTGTCTTCTGAACGTC	CGTCCGATCAACATGCTCACT
*Nusap1*	CGTCACCAAAACGAGGAGGAG	AGAAAACTCATCCGTGCATAGAG
*GAPDH*	TGTGTCCGTCGTGGATCTGA	TTGCTGTTGAAGTCGCAGGAG

### 2.6 Western blot Analysis

The details of Western blot analysis are described in Supporting Information.

### 2.7 Enzyme‐Linked Immunosorbent Assay

Cell culture supernatants were analyzed using a mouse IL‐1β ELISA kit (CME0015; 4A Biotech, Beijing, China), according to the manufacturer's instructions.

### 2.8 Statistical Analysis

GraphPad Prism 9.4 and R software v. 3.6.3 were used for statistical analysis, and results were obtained from at least three independent experiments. Data are expressed as the means ± standard deviation, and an unpaired Student's *t*‐test or univariate analysis of variance was used for comparison between groups. A *p* value < 0.05 was considered to be indicative of statistical significance.

## 3 Results

### 3.1 Proteomic Characterization and Identification of Proteins

**Figure 2:**
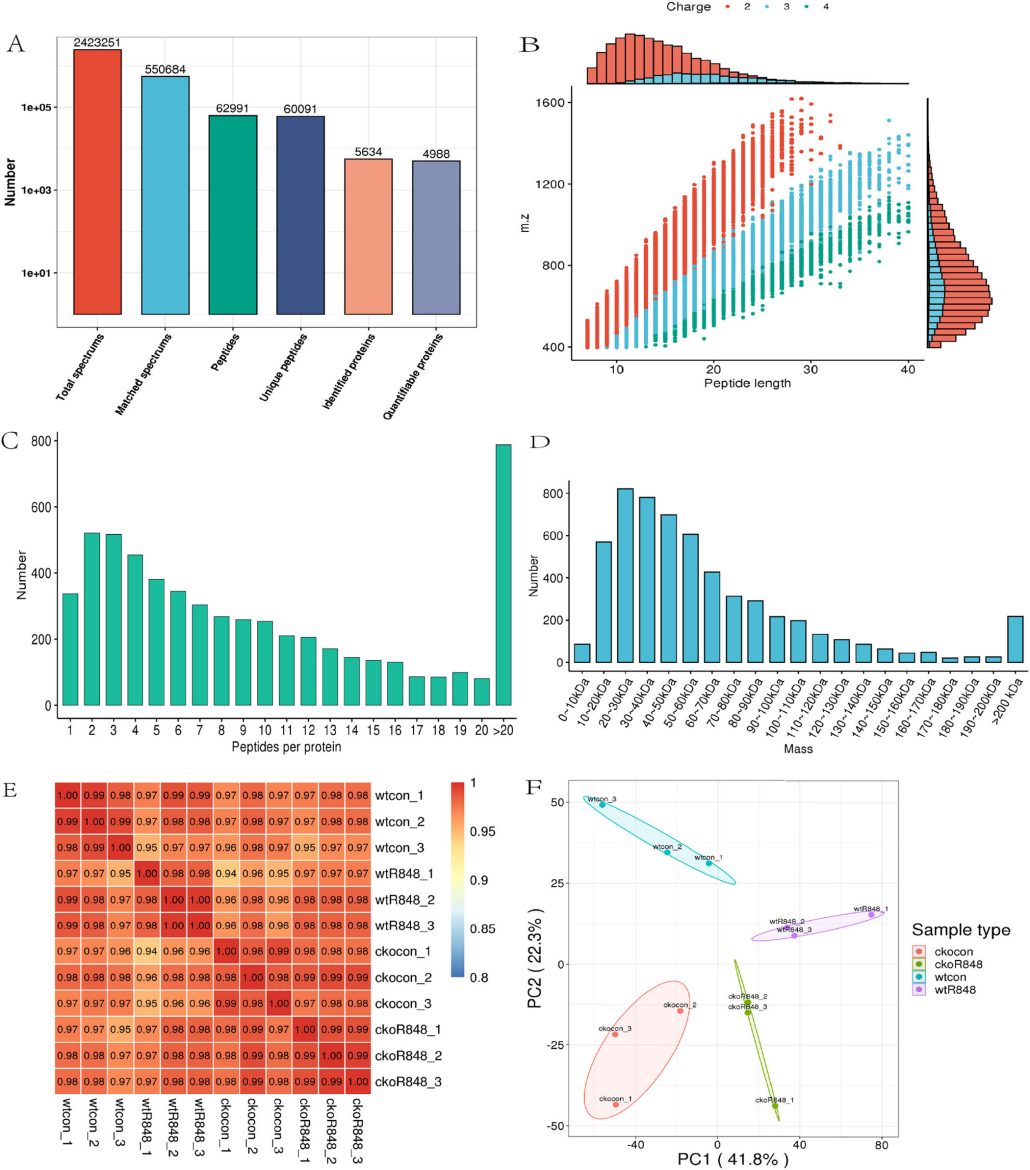
Proteomic characterization and identification of proteins. (A) Overview of protein identification. (B) Peptide length distribution diagram. (C) Map of peptides per protein distribution. (D) The molecular weight distribution of proteins. (E) Heatmap of Pearson's Correlation Coefficient (PCC). A PCC close to −1 indicates a negative correlation, close to 1 indicates a positive correlation, and close to 0 indicates no correlation. (F) Principal component analysis (PCA) diagram. The degree of aggregation among samples in the figure represents the difference of samples.

In total, 5634 proteins were identified, among which 4988 were quantified based on 62,991 unique peptides detected in an MS/MS spectrum database search (Figure 2A). Most peptides ranged in size from 7 to 20 amino acids, consistent with the enzymatic hydrolysis preparation step and MS fragmentation mode (Figure 2B). Furthermore, most proteins comprised more than two peptides (Figure 2C), and the coverage of the majority was > 30%. In the shotgun‐based MS analysis, peptides with a higher abundance were preferentially scanned, resulting in a positive correlation between protein coverage and abundance. The molecular weights of the identified proteins were determined at different stages and evenly distributed, and the length distribution of the peptides met the quality control requirements (Figure 2D). In total, 4716 proteins were annotated to Clusters of Orthologous Groups of proteins/EuKaryotic Orthologous Groups, 4274 to GO, 2872 to KEGG, and 3176 to Pftam databases. Both Pearson correlation and principal component analyses revealed the quantitative reproducibility of the protein samples across the three replicates for each groups (Figure 2E,F).

### 3.2 Identification of DEPs in BMDM Samples

**Figure 3:**
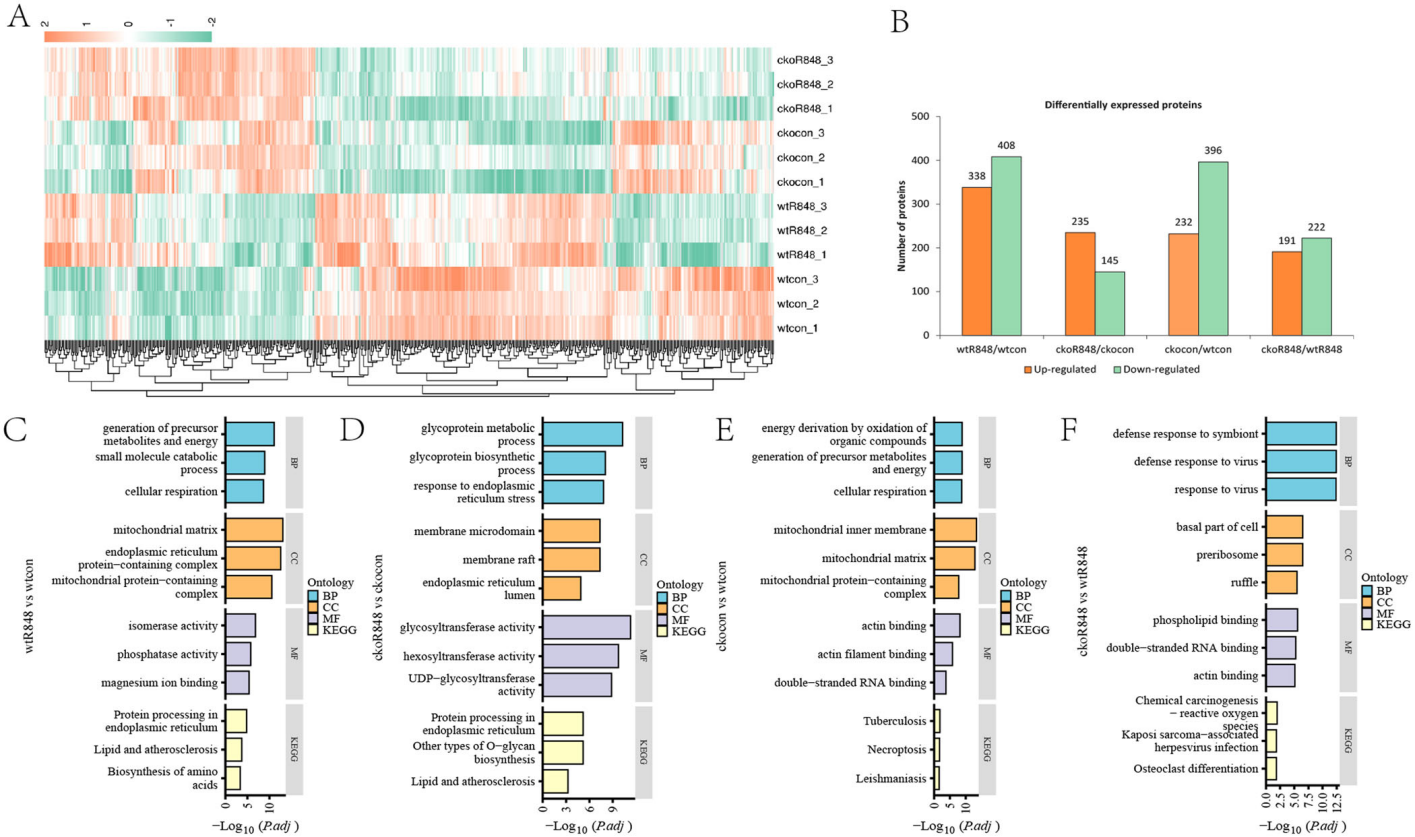
Identification of differentially expressed proteins (DEPs) in bone marrow‐derived macrophage samples. (A) Heatmap of DEP expression. (B) Bar chart of DEPs in each comparison group. (C–F) Bar chart of GO and KEGG analysis (*n* = 3).

Differential protein analyses of four binary comparison groups (wtcon, wtR848, ckocon, and ckoR848) were performed to further investigate the mechanisms underlying *Pggt1b* deficiency‐induced macrophage activation in R848‐stimulated BMDMs. The criteria for significant differences between the two groups were log FC ≥ 1.3 and *p* < 0.05. A corresponding heatmap and the up‐ and downregulated proteins are displayed in a histogram (Figure 3A,B). When comparing proteins differentially expressed in wtR848 and wtcon, we found that DEPs enriched in the GO category BP are associated with the generation of precursor metabolites, small‐molecule catabolic process, and cellular respiration. In CC enrichment, DEPs were notably associated with the mitochondrial matrix, endoplasmic reticulum protein‐containing complex, and mitochondrial protein‐containing complex, whereas in the MF category, they were enriched in isomerase activity, magnesium ion binding, and manganese ion binding. In addition, KEGG pathway analysis revealed enrichment in the biosynthesis of amino acids and protein processing in the endoplasmic reticulum (Figure 3C).

In the ckoR848 versus ckocon comparison, the top three most markedly enriched BP terms were glycoprotein metabolic process, glycoprotein biosynthetic process, and response to endoplasmic reticulum stress, whereas the top three CC terms were membrane microdomain, membrane raft, and endoplasmic reticulum lumen, and the top three MF terms were glycosyltransferase activity, hexosyltransferase activity, and UDP‐glycosyltransferase activity. Furthermore, KEGG pathway analysis revealed the DEPs to be primarily enriched in protein processing in the endoplasmic reticulum, followed by other types of processes such as *O*‐glycan biosynthesis and glycosaminoglycan biosynthesis (Figure 3D).

In the ckocon versus wtcon comparison, energy derivation by oxidation of organic compounds, generation of precursor metabolites, and cellular respiration were enriched in BP terms, whereas the top four CC enriched terms were mitochondrial inner membrane, mitochondrial matrix, organelle inner membrane, and mitochondrial protein complex, and the top three enriched MF terms were actin binding, actin filament binding, and double‐stranded RNA binding (Figure 3E). With respect to DEPs in the ckoR848 versus wtR848 comparison, the most significantly enriched GO terms in BP were the defense response to the symbiont and defense response to the virus, whereas the most significantly enriched GO terms in CC were basal part of cell and preribosome, and among MF terms, they were enriched in phospholipid binding, actin binding, and double‐stranded RNA binding. Furthermore, the most enriched KEGG pathway was chemical carcinogenesis‐reactive oxygen (Figure 3F). These results indicate that in response to stimulating BMDMs with R848, mitochondrial transferase activity was induced to activate protein processing in the endoplasmic reticulum. Furthermore, *Pggt1b* deficiency in BMDMs altered the capacity of these cells to regulate innate immune responses and enhanced mitochondrial function in generating precursor metabolites and energy. When stimulated with R848, *Pggt1b‐*deficient BMDMs were more readily induced to mount defense responses to symbionts and viruses.

### 3.3 Pggt1b Positively Regulates Cytokine Production in R848‐Stimulated BMDMs

**Figure 4:**
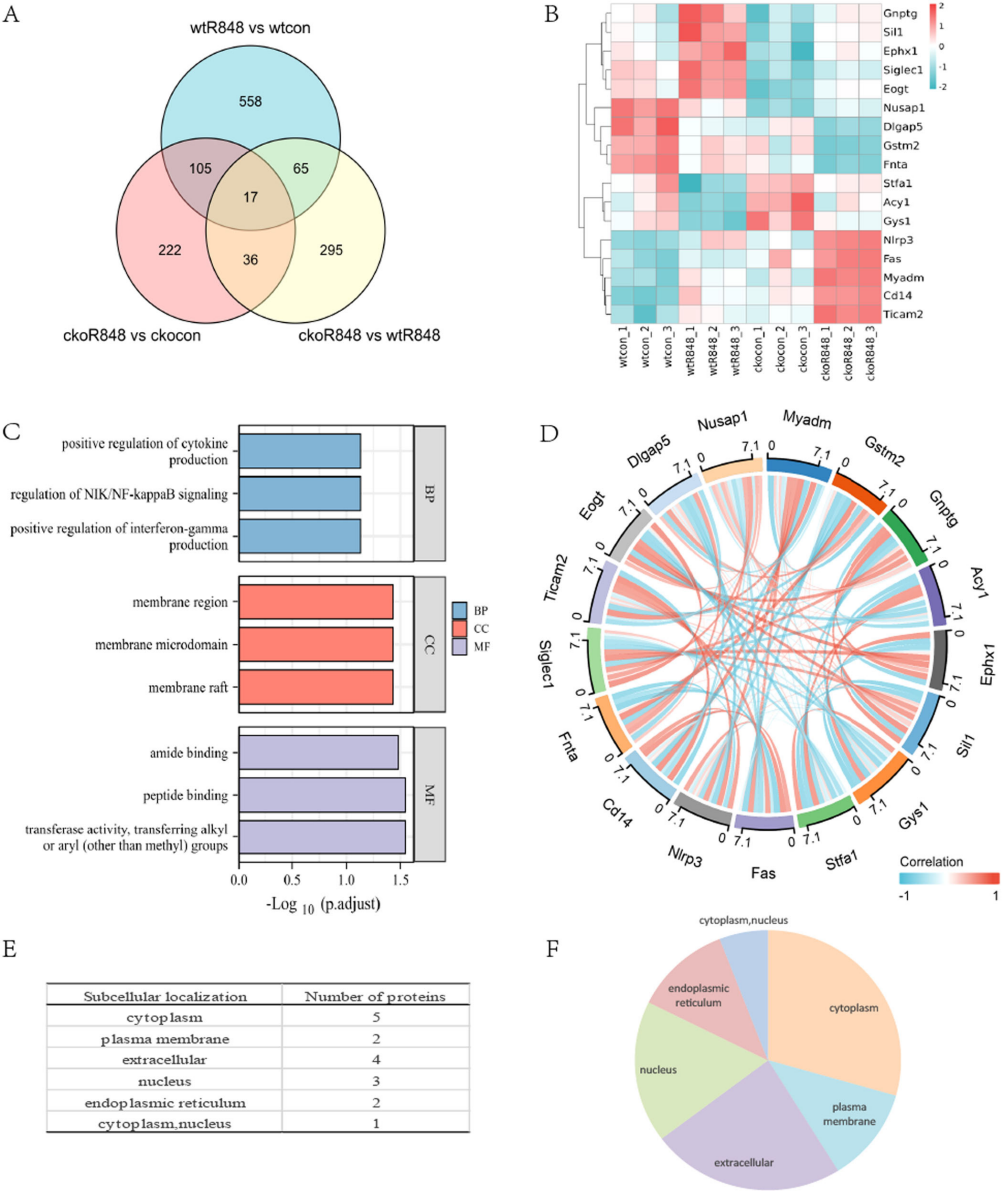
*Pggt1b*‐positive regulation of the cytokine production in R848‐stimulated bone marrow‐derived macrophages. (A) Venn diagrams showing the number of unique and shared DEPs. (B) Heatmap of hub proteins in liquid chromatography–tandem mass spectrometry analysis. (C) Bar graph showing Gene Ontology (GO) enrichment analysis of hub proteins. (D) String diagram of hub protein interactions. (E and F) Subcellular localization of hub proteins.

**Figure 5:**
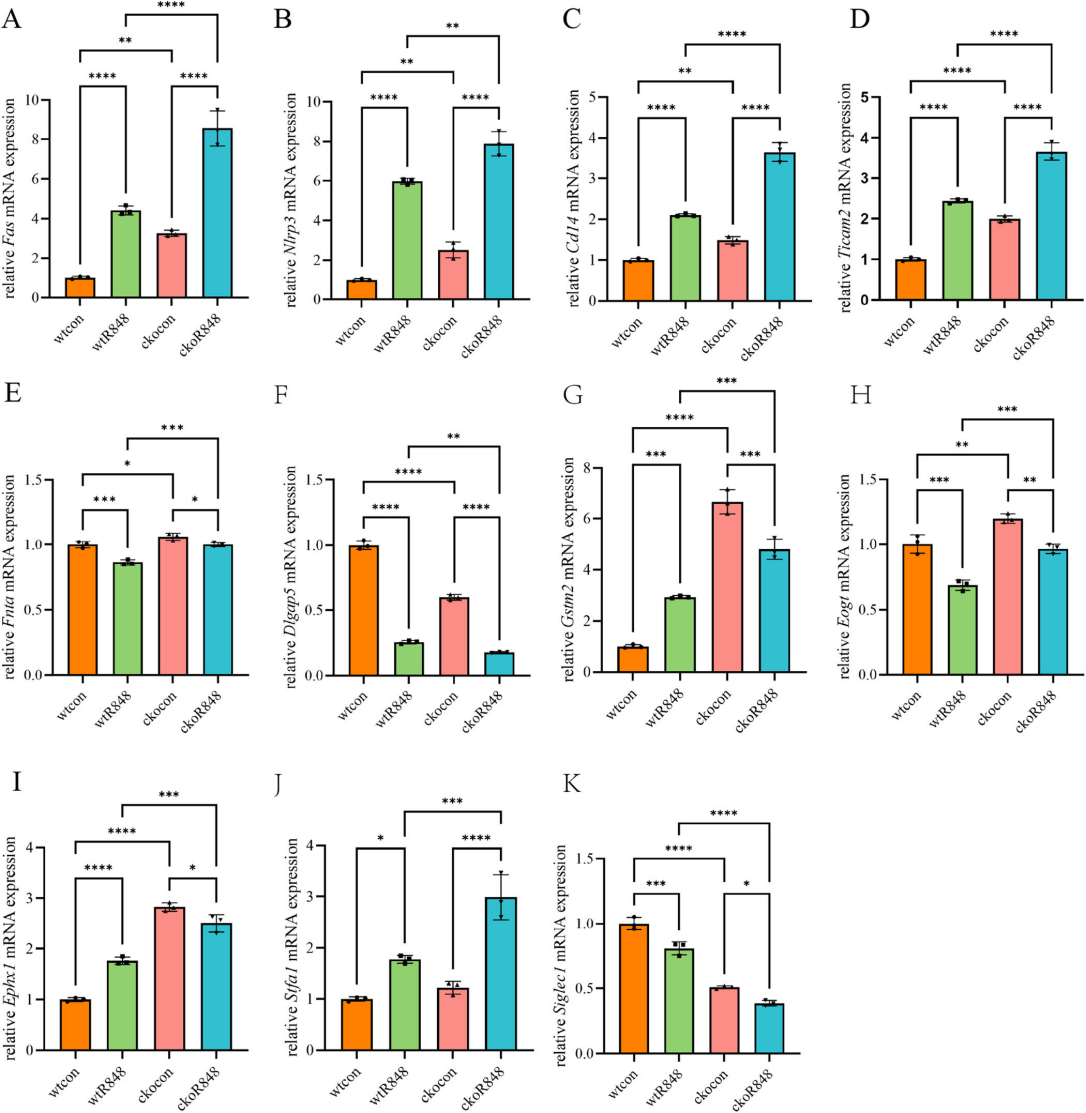
qRT‐PCR analysis of mRNA candidate proteins normalized to GAPDH mRNA expression. (A–F) mRNA expression difference is consistent with that of the protein. (G–K) mRNA and protein expression trends are not completely consistent (*p < 0.05; **p < 0.01; ***p < 0.001; ****p < 0.0001). The data represent the mean ± SD of three independent experiments (*N* = 3, *n* = 1), where each repetition involved the use of BMDM from one mouse per group.

We performed an LC‐MS/MS analysis to identify DEPs in the wtR848 versus wtcon, ckoR848 versus ckocon, and ckoR848 versus wtR848 groups, to gain a better understanding of how *Pggt1b* influences the response of BMDMs to R848 stimulation. We identified 17 DEPs (Table 2, Figure 4A), a heatmap for which is shown in Figure 4B. The top three significantly enriched GO terms in the BP category were positive regulation of cytokine production, NF‐κB inducing kinase/NF‐κB signaling regulation, and positive interferon (IFN)‐γ production regulation, whereas those in the CC category were membrane region, microdomain, and raft. The top four enriched MF terms were amide binding, peptide binding, transferase activity, and transferring alkyl or aryl (other than methyl) groups (Figure 4C). Figure 4D presents a chord diagram of the protein interactions. We also analyzed the subcellular localization of the 17 DEPs, which accordingly revealed that Gstm2, Stfa1, Fnta, Ticam2, and Acy1 were located in the cytoplasm; Siglec1 and Myadm were detected in the plasma membrane; Cd14, Fas, Gnptg, and Sil1 were predominant in the extracellular region; disks large‐homolog‐associated protein 5 (Dlgap5), Nusap1, and Gys1 were localized to the nucleus; and Eogt and Ephx1 were present in the endoplasmic reticulum. In addition, Nlrp3 was established to be located in both the cytoplasm and the nucleus (Figure 4E,F). The DEPs were validated through qRT‐PCR analysis, which indicated that differences in mRNA expression of six proteins were consistent with those of the reference proteins (Figure 5A–F). These six DEPs (Dlgap5, Fas, Fnta, Nlrp3, Cd14, and Ticam2) were defined as hub proteins, representing five of the mRNA expression trends of the DEPs. The expression of these protein was not completely consistent with the corresponding mRNA expression (Figure 5G–K). Moreover, we detected no significant difference among the six DEPs with respect to mRNA expression (Figure S1).

**Table 2:** Proteins differently expressed in wtR848 versus wtcon, ckoR848 versus ckocon, and ckoR848 versus wtR848.

Protein accession	Gene name	Protein description
O35682	*Myadm*	Myeloid‐associated differentiation marker
P15626	*Gstm2*	Glutathione S‐transferase Mu 2
Q6S5C2	*Gnptg*	*N*‐acetylglucosamine‐1‐phosphotransferase subunit γ
Q99JW2	*Acy1*	Aminoacylase‐1
Q9D379	*Ephx1*	Epoxide hydrolase 1
Q9EPK6	*Sil1*	Nucleotide exchange factor SIL1
Q9Z1E4	*Gys1*	Glycogen [starch] synthase, muscle
P35175	*Stfa1*	Stefin‐1
P25446	*Fas*	Tumor necrosis factor receptor superfamily member 6
Q8R4B8	*Nlrp3*	NACHT, LRR, and PYD domains‐containing protein 3
P10810	*Cd14*	Monocyte differentiation antigen CD14
Q61239	*Fnta*	Protein farnesyltransferase/geranylgeranyltransferase type‐1 subunit α
Q62230	*Siglec1*	Sialoadhesin OS= *Mus musculus*
Q8BJQ4	*Ticam2*	TIR domain‐containing adapter molecule 2
Q8BYW9	*Eogt*	EGF domain‐specific *O*‐linked *N*‐acetylglucosamine transferase
Q8K4R9	*Dlgap5*	Disks large‐associated protein 5
Q9ERH4	*Nusap1*	Nucleolar and spindle‐associated protein 1

### 3.4 Pggt1b Deficiency Influences NF‐κB Activation

**Figure 6:**
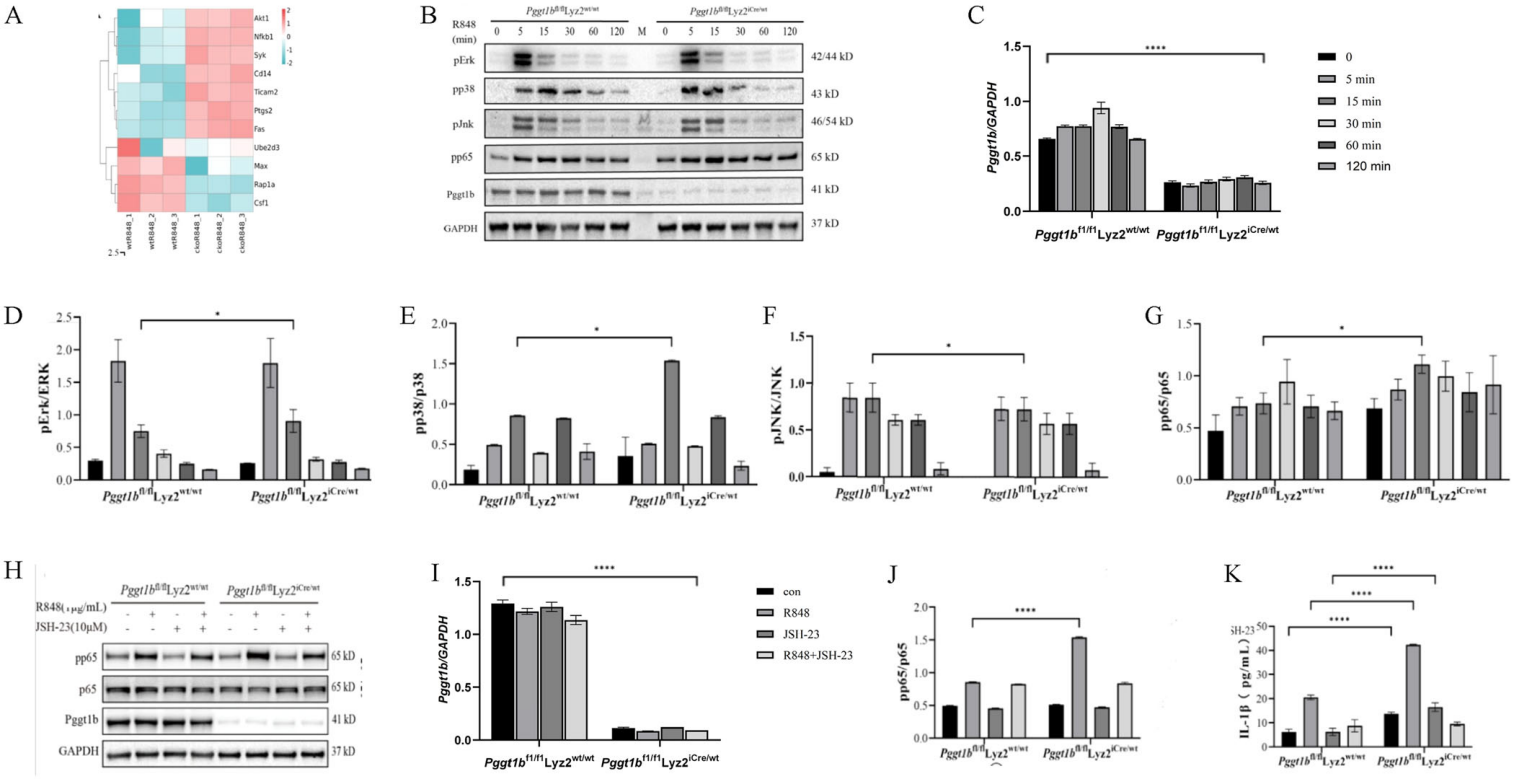
The nuclear factor‐κB (NF‐κB) pathway is enhanced in Pggt1b‐deficient BMDMs. (A) Heatmap showing the expression of core proteins that contribute to NF‐κB pathway enrichment (pink, high abundance; blue, low abundance). (B) Immunoblot analysis of phosphorylated p65 and Pggt1b in BMDM lysates stimulated (above lanes) with R848 (1 μg/mL). (C–G) Statistical analysis of interested protein levels was shown (*p < 0.05). The data represent the mean ± SEM of three independent experiments. (H) Immunoblot analysis of phosphorylated p65 and p65 in BMDM lysates stimulated with or without R848 (1 μg/mL) and JSH‐23 (10 μM). (I‐J) Statistical analysis of the levels of proteins of interest (*p < 0.05; **p < 0.01; ***p < 0.001; ****p < 0.0001). The data represent the means ± SEM of three independent experiments. (K) Interleukin (IL)‐1β concentrations in the supernatants of cultured wild‐type and Pggt1b‐deficient BMDMs stimulated with or without R848 (1 μg/mL) and JSH‐23 (10 μM), determined using enzyme‐linked immunosorbent assay (ELISA). (*p < 0.05; **p < 0.01; ***p < 0.001; ****p < 0.0001). The data represent the mean ± SD of three independent experiments (*N* = 3, *n* = 1), where each repetition involved the use of BMDM from one mouse per group.

The KEGG enrichment analysis indicated that proteins in the NF‐κB and Ras signaling pathways were more abundant in ckoR848 compared with those in wtR848 (Figure 6A). Given the altered cytokine production in *Pggt1b*‐deficient macrophages, we evaluated the activation of MAPK and NF‐κB signaling pathways downstream of TLRs. Wild‐type and *Pggt1b*‐deficient BMDMs were stimulated with R848 at different time points, and we determined the activation of Erk, p38, JNK, IκBα, and NF‐κB. Whereas the activation of JNK and Erk was unaffected, we detected an enhancement of p38, IκB, and NF‐κB activation in the *Pggt1b*‐deficient macrophages than that in the wild‐type control cells (Figure 6B–G, Figure S2). We thus speculate that the altered cytokine profile of the *Pggt1b*‐deficient macrophages could be attributable to altered p38, IκB, and TLR endocytosis activation. Subsequently, we assessed the effects of treatment with JSH‐23 (HY‐13982, MCE; 10 μmol), an NF‐κB inhibitor that inhibits NF‐κB transcriptional activity without degrading IκBα, and accordingly observed that in the *Pggt1b*‐deficient BMDMs, NF‐κB activation was reduced to almost the same level as that in the wild‐type BMDMs (Figure 6H–J). In addition, our detection of secreted IL‐1β in the BMDM supernatant confirmed that *Pggt1b* knockout promotes inflammatory cytokine secretion via the NF‐κB pathway. Moreover, R848 significantly promoted IL‐1β secretion in *Pggt1b*‐deficient macrophages, whereas secretion was reduced in JSH‐23‐treated cells (Figure 6K). These findings indicate that *Pggt1b*‐deficient macrophages are activated and are more likely to produce large amounts of IL‐1β when stimulated.

## 4 Discussion

Psoriasis is a chronic inflammatory skin disease that affects 2%–3% of the global population, and accordingly constitutes a significant global burden. Recent studies have demonstrated that psoriatic skin inflammation is a source of inflammatory reactions involved in a range of systemic inflammatory disorders. For example, patients with psoriasis have a particularly heightened risk of cardiovascular disease [16] and bone fractures [17]. At present, it is believed that psoriasis is an immune abnormality induced by genetic and environmental factors, involving keratinocytes and various immune cells, which together form an inflammatory circuit and promote the onset and development of psoriasis [18]. Therefore, the level of inflammatory factors is closely related to the onset and severity of psoriasis, among which IL‐17 and IL‐23 play a key role. Both cytokines are related to the severity and persistence of the disease. It was found that the levels of IL‐17 and IL‐23 are consistently elevated in the skin of psoriatic patients. IL‐17 expression was significantly increased in diseased skin compared with non‐diseased skin and healthy controls [19]. In addition, the level of IL‐17A in the circulating blood of patients with psoriasis also increased [20]. Similarly, IL‐23, as a key upstream regulator of IL‐17 production, is also upregulated in psoriasis skin [21]. It was shown that IL‐23 mRNA and protein levels significantly increased in diseased skin, especially in dendritic cells and macrophages [22]. IL‐23 can amplify the inflammatory response by promoting the differentiation and proliferation of Th17 cells. Th17 cells are a special kind of T cells, which can secrete a lot of IL‐17 and other inflammatory cytokines. These cells can stimulate the production of a variety of antimicrobial peptides, chemokines, and factors that promote inflammation and cell proliferation, thus aggravating the inflammatory reaction in tissues [23, 24]. In animal models of psoriasis, such as psoriasis‐like dermatitis induced by IMQ in mice, macrophages treated with IL‐23 significantly promote the onset of dermatitis in psoriasis‐like mouse models [25]. In addition, T cells with high levels of IL‐17 produce self‐amplified precursor inflammatory reactions in keratinocytes, which can drive the development of thickened skin lesions [26]. Meanwhile, biological agents that currently inhibit TNF‐α, p40, IL‐12/23, and IL‐17 have also been approved for psoriasis treatment [27, 28]. However, although these agents have been established to have beneficial therapeutic effects, the relapse rate of psoriasis remains high. Challenges to administering effective treatment stem from the complex pathophysiology, diverse treatment responses among patients, and the difficulty in achieving long‐term remission. Consequently, developing more effective treatments and designing personalized therapeutic strategies remains a key focus for future research and psoriasis management in clinical practice. In this context, a growing body of research has indicated that macrophages play pivotal roles in inducing and promoting the progression of psoriatic inflammation, and pro‐inflammatory activated macrophages can cause psoriasis inflammation [29].

The expression of *PGGT1B* has been established to decline in the peripheral blood mononuclear cells of patients with psoriasis, and the downregulation of PGGT1B in PBMCs may increase the expression of pro‐inflammatory cytokines and consequentially activate the inflammatory signaling that contributes to exacerbating the severity of psoriasis [9]. Notably, PGGT1B is expressed in a range of different cell types, including the monocytes of peripheral blood cells, digestive tract epithelial cells, endocrine gland glandular cells, and skin cells, and PGGT1B deficiency has been linked to the development of chronic inflammatory diseases. To further explore the mechanism of *PGGT1B* in psoriasis, we adopted the model of BMDMs stimulated by R848 and found that *PGGT1B* deficiency led to a series of expression changes of inflammation‐related genes, including the upregulation of IFN‐β, which was different from previous studies using poly I:C as stimulator, suggesting that *PGGT1B* may affect the inflammatory response through different pathways under different stimulation modes [30]. Based on our experimental data, we speculate that Pggt1b deficiency may promote the secretion of inflammatory cytokines, including IL‐1, IL‐17, and type I IFN, by activating the NF‐κB signaling pathway, which echoes the previous findings in the detection of psoriasis skin mRNA [9]. In particular, IL‐1α, as the main form of IL‐1 activity in skin tissue, directly affects the growth and differentiation of keratinocytes through its combination with IL‐1R1, which is closely related to the pathological features of psoriasis. At the same time, IL‐1β, as the main inducer of acute reaction, not only regulates cellular immune response but also promotes T cell proliferation and IL‐17 production, which plays a central role in the pathogenesis of psoriasis [23, 31].

mRNA translation to proteins involves several regulatory steps, including posttranscriptional modification, RNA splicing, RNA transport, and posttranslational modification, all of which might influence protein expression and activity. Discrepancies between mRNA and protein levels can be attributed to differences in posttranscriptional modifications and splicing, regulation of mRNA stability, variations in translation rate and efficiency, and the complex regulatory factors within the cellular environment. These factors collectively contribute to the divergence of transcriptional and translational processes in terms of quantity and efficiency, ultimately affecting the final levels of protein expression [32]. In the present study, as candidate proteins, we selected only those proteins for which the expression trends were consistent with those of mRNAs. We accordingly identified a total of 17 DEPs associated with *Pggt1b* deficiency in R848‐stimulated BMDMs, leading us to speculate on the potential mechanisms whereby R848 promotes inflammation. We also established that mRNA expression of the six hub proteins Dlgap5, Fas, Fnta, Nlrp3, Cd14, and Ticam2 was influenced by *PGGT1B*. Nonetheless, a limitation of this study is that we did not verify differential protein expression at the protein level.

Geranylgeranylation is a lipoprotein modification that involves small‐monomer GTP‐binding proteins and plays a key role in the functional activation of small G proteins by regulating membrane localization. Ticam2, also referred to as TIR‐domain‐containing adapter molecule 2, forms a complex with RAB11FIP2, which is recruited to phagosomes to promote activation of the actin‐regulatory GTPases RAC1 and CDC42, leading to the phagocytosis of gram‐negative bacteria [33]. Both in vivo and in vitro studies (knockout mice and organoids) have identified RAC1 as a GGTase target intimately involved in prenylation‐dependent cytoskeleton dynamics, cell mechanics, and epithelial cell shedding [34]. In this regard, we suggest that *Pggt1b* might mediate R848‐induced inflammation via the small‐G protein Rac1 or Cdc42. Fnta is an essential subunit of the farnesyltransferase and geranylgeranyltransferase complexes, and FNTA‐siRNA and EHop have been shown to inhibit glucose‐induced activation of Rac1–Nox2–ROS signaling in primary human retinal endothelial cells [35]. The canonical TLR pathway responsible for the R848‐mediated effects may contain a protein component that is heavily dependent on farnesylation for adequate signaling. These findings indicate that in the absence of *Pggt1b*, the function of Fnta is enhanced to compensate for the changes in biological function caused by geranylgeranyl deletion. However, Diehl et al. have observed the simultaneous upregulation of FNTA and PGGTIB in response to LPS (TLR2/4) stimulation in gingival fibroblasts, although identified no putative targets to support this hypothesis [36]. Our results suggest that the canonical TLR pathway responsible for the response to LPS contains a protein component that is dependent on farnesylation for adequate signaling.

In response to a deficiency in *Pggt1b*, we detected a significant increase in *Fas* expression following R848 stimulation, which is consistent with findings reported in the existing literature. Notably, *Fas* upregulation may occur during new‐onset psoriasis due to lymphocyte activation, and this upregulation is necessary for inducing key inflammatory cytokines, including TNF‐α and IL‐15 [37, 38]. Although *Fas* (CD95) activation typically induces apoptosis, an alternative pathway of Fas signaling induces inflammatory cytokines. Acquavella et al. found that treatment with simvastatin heightened the susceptibility of liver sinusoidal endothelial cells mediated by *Fas*, which is inconsistent with our findings in this study. In contrast to our observation, these authors failed to detect any elevation in *Fas* levels [39]. We suspect that this disparity could be associated with a difference in cell type, endothelial cells in their study, and immune cells in the present study. Alternatively, this could be ascribed to the fact that simvastatin inhibits mevalonate synthesis while also inhibiting geranylgeranylation of the protein [40] and that alterations in the process of mevalonate synthesis or in its content influence Fas expression. Notably, *Fas* overexpression provided evidence suggesting that *Pggt1b* knockout may promote an increase in inflammatory cytokines during the initial stages of psoriasis.

Although we have found important findings in this study, it also has some limitations. First, the macrophages used in our experiment are derived from mice with specific gene knockout and stimulated by TLR7/8 agonists, instead of directly using psoriasis‐specific models. After TLR7/8 is activated, it will trigger a series of downstream signal transduction events, including the activation of NF‐kB, MAPK, and IRF [11, 41]. The activation of these pathways not only promotes the production of inflammatory cytokines, such as TNF‐α, IL‐6, and IL‐1β, but also induces the maturation and migration of immune cells [42, 43]. In the skin lesions of patients with psoriasis, the activities of these cytokines and immune cells are also increased, and they interact to form a complex inflammatory network, which drives the occurrence and development of psoriasis. At the same time, TLR7/8 activation can also simulate the excessive proliferation and abnormal differentiation of keratinocytes in psoriasis. In the pathological process of psoriasis, the proliferation of keratinocytes is accelerated under the stimulation of inflammation, accompanied by abnormal differentiation procedures. After TLR7/8 is activated, the proliferation and differentiation of keratinocytes can be induced by releasing inflammatory cytokines and IFN, thus simulating psoriasis‐like epidermal changes in vitro [11, 44]. It is worth noting that although TLR7/8 activation can simulate many aspects of psoriasis inflammation, it does not mean that it is the only or fundamental cause of psoriasis. Therefore, in future research, we will further use the psoriasis‐specific model (e.g., IMQ‐induced psoriasis in vivo) to confirm the correlation between these findings and psoriasis. Secondly, we did not identify the hub protein by western blot analysis, so we may have missed the protein whose mRNA and protein expression are inconsistent. Thirdly, although the protein profile was used in this study, lipoprotein modification was not analyzed, and the activities of RAC1 and CDC42 were not determined. In addition, the experimental results obtained in this study are only confirmed at the cellular level, and further experiments may be needed to establish the mechanism of *PGGT1B* deletion to further verify our experimental results in vivo. These are all areas that we intend to focus on in future research.

## 5 Conclusions

In conclusion, our findings in this study indicate that a deficiency in *Pggt1b* is associated with an alteration in the regulation of the innate immune response and enhances mitochondrial function, resulting in the generation of precursor metabolites and energy in R848‐stimulated BMDMs. These findings indicate that in *Pggt1b*‐deficient macrophages, TLR4 might be in a highly activated state and is activated by R848 or damage‐associated molecular patterns. This was found to be closely correlated with an increase in cytokine production, IFN‐γ production, amide binding and transferase activity augmentation, and NF‐κB and Nlrp3 inflammasome pathway activation, which plausibly lead to hyperinflammatory responses in *Pggt1b* ^fl/fl^Lyz2^iCre/wt^ mice, although this was not observed in *Pggt1b* ^fl/fl^Lyz2^wt/wt^ mice. These findings have significant implications for elucidating the pathogenesis of macrophages in inflammatory responses when stimulated with R848. In this study, we performed a preliminary analysis of the effects of *Pggt1b* on innate immunity, the findings of which provide a theoretical basis for treating clinical inflammatory diseases such as psoriasis. We anticipate further important discoveries regarding the activity of Pggt1b in inflammation, which will undoubtedly yield further insights for future clinical practice.

## Author Contributions

Shanshan Yu designed the study and wrote the main manuscript. Xuecui Wei and Fangyuan Long collected the data, carried out data analyses, and produced the initial draft of the manuscript. Zhimin Hao and Heng Gu funded the acquisition and contributed to the draft of the manuscript. All authors have reviewed the manuscript and agreed to the published version of the manuscript.

## Ethics Statement

The animal procedures performed in this study were approved by The Ethics Committee of Dermatology Hospital, Chinese Academy of Medical Sciences (approval no. 22‐DW‐007).

## Conflicts of Interest

The authors declare no conflicts of interest.

## Supporting information


**Fig**. **S1**. Quantitative real‐time PCR analysis of mRNA candidate proteins normalized to the expression of GAPDH mRNA levels. (*, *P* < 0.05; **, *P* < 0.01; ****, *P* < 0.0001). **Fig**. **S2**. NF‐κB pathway is enhanced in Pggt1b‐deficient BMDMs. (A) Western blot analysis of phosphorylated IκBα, IκBα, and Pggt1b in BMDM lysates stimulated (above lanes) with R848 (1 μg/mL). (B)‐(D) Statistical analysis of interested protein levels was shown. GAPDH served as a loading control.(*, *P* < 0.05; **, *P* < 0.01; ***, *P* < 0.001; ****, *P* < 0.0001). The data represent the mean ± SD of three independent experiments (N=3, n=1), where each repetition involved the use of BMDM from one mouse per group.

## Data Availability

The datasets used and/or analyzed during the current study are available from the corresponding author upon reasonable request.
