# Supplementary material for: Quantitative Proteomic Analysis Indicates That Pggt1b Deficiency Promotes Cytokine Secretion in Resiquimod‐Stimulated Bone Marrow‐Derived Macrophages via the NF‐κB Pathway
Source: Immun Inflamm Dis. 2025 Apr 7;13(4):e70185. doi: 10.1002/iid3.70185 (PMC11973730; doi:10.1002/iid3.70185)
Supplement: Supplementary file 1 — Fig. S1. Quantitative real‐time PCR analysis of mRNA candidate proteins normalized to the expression of GAPDH mRNA levels. (*, P < 0.05; **, P < 0.01; ****, P < 0.0001). Fig. S2. NF‐κB pathway is enhanced in Pggt1b‐deficient BMDMs. (A) Western blot analysis of phosphorylated IκBα, IκBα, and Pggt1b in BMDM lysates stimulated (above lanes) with R848 (1 μg/mL). (B)‐(D) Statistical analysis of interested protein levels was shown. GAPDH served as a loading control.(*, P < 0.05; **, P < 0.01; ***, P < 0.001; ****, P < 0.0001). The data represent the mean ± SD of three independent experiments (N=3, n=1), where each repetition involved the use of BMDM from one mouse per group. [file IID3-13-e70185-s001.docx]

**Supplementary Information**

**Supplementary materials and methods**

**BMDM culture and stimulation**

Bone marrow cells were collected from wild-type and conditional knockout mice aged 6-8 weeks by washing femur and tibia with phosphate buffered saline (PBS). Firstly, bone marrow cells were inoculated into a 75 cm² culture flask (Sarstedt, Nümbrecht, Germany) at a density of 5× 10^5^ cells /mL, and 20 mL of Dulbecco's modified eagle medium (DMEM) (10270106; Gibco) and 20% L929 cell conditioned medium (L929 supernatant, ATCC, Manakas, Virginia). During the initial 7-day differentiation period, the culture medium was not changed to ensure consistent differentiation conditions. Flow cytometry analysis showed that bone marrow cells were effectively differentiated into bone marrow-derived macrophages (BMDMs) after staining with CD11b and F4/80 antibodies. At the end of the 7-day differentiation period, the cells were transferred from the culture flask to a 6-well tissue culture plate. Enzymatic treatment (trypsin) is usually used in the transfer process to digest the cells and make them fall off the surface of the culture bottle. The specific operation is: treat the cells with an appropriate amount of enzyme solution, gently shake them to promote cell shedding, then neutralize the enzyme activity and collect the cells. The collected cells were resuspended in DMEM supplemented with 10% fetal bovine serum and inoculated into 6-well plates (Corning, new york, USA) at a density of 1× 10^6^ cells/well, and 2 mL of the above DMEM medium containing 10% FBS and 20% L929 supernatant was added to each well. Subsequently, BMDMs in 6-well plates were mixed with 1 μg/mL R848(S28463; MedChemExpress, Monmouth Junction, NJ, USA) or dimethyl sulfoxide (DMSO, D2650; Sigma-Aldrich, St. Louis, Missouri, USA). The viability of BMDM was evaluated by annexin V/ propidium iodide staining, followed by flow cytometry. We observed that about 85% of the cells were still alive after treatment, indicating that the cytotoxic effect was minimal at this dose. The experiment comprised the following four treatment groups: wild-type BMDMs stimulated with DMSO (wtcon), wild-type BMDMs stimulated with R848 (wtR848), *Pggt1b*-deficient BMDMs stimulated with DMSO (ckocon), and *Pggt1b*-deficient BMDMs stimulated with R848 (ckoR848). After 5 h, the cells were collected for liquid chromatography–tandem mass spectrometry (LC-MS/MS) and quantitative real-time analyses. In addition, BMDMs were collected at 0, 5, 15, 30, 60, and 120 min post-stimulation for signaling pathway validation via western blot analysis.

**LC-MS/MS analysis**

A high-intensity ultrasonic processor (JY92-IIN; Scientz,Ningbo, Ningbo, China) was used to process the samples in a lysis buffer containing 8 M urea and a 1% protease inhibitor cocktail. The remaining debris was removed via centrifugation at 12,000 × *g* for 10 min at 4°C. The supernatant protein concentration was determined using a bicinchoninic acid (BCA) kit (P0010; Beyotime, Jiangsu, China). To obtain the protein solution, samples were reduced with 5 mM dithiothreitol for 30 min at 56°C and alkylated with 11 mM iodoacetamide (V900335, Sigma-Aldrich) for 15 min at 25°C in the dark. The protein samples were then diluted using triethylammonium bicarbonate buffer (140023; Sigma-Aldrich) to a urea concentration of less than 2 M, followed by the addition of trypsin at 1:50 for 1 day of digestion and at 1:100 for subsequent 4 h. Finally, the peptides were desalted using a C18 SPE column (8B-S100-AAK; Phenomenex, Torrance, CA, USA). Solvent A (0.1% formic acid and 2% acetonitrile in water) was used to dissolve the tryptic peptides, which were directly injected into a reversed-phase analytical column (25 cm long and 75/100 μm in diameter). The peptides were separated using a gradient of solvent B (0.1% formic acid in acetonitrile) from 6% to 24% for 70 min, 24% to 35% for 14 min, then increasing to 80% in 3 min, followed by holding at 80% for the final 3 min, all at a constant flow rate of 450 mL/min in a nanoElute ultra-high-performance liquid chromatography system (Bruker, Billerica, MA, USA). A 1.75-kV electrospray voltage was used to electrospray the peptides after mass spectrometry using a timsTOF Pro mass spectrometer (Bruker). The precursors and fragments were analyzed using a time-of-flight detector, with an MS/MS scan range of 100–1700 m/z. For each precursor with a charge state of 0–5A, ten PASEF-MS/MS scans were obtained using timsTOF Pro in the parallel accumulation mode. The dynamic exclusion was set to 30 s. The resulting MS/MS data were processed using the MaxQuant search engine (v. 1.6.15.0). Tandem mass spectra were searched against Mus_musculus_10090_SP_20220107.fasta (17097 entries) concatenated with a reverse decoy database. Trypsin/P was specified as the cleavage enzyme, which permits up to two missed cleavages. The primary and secondary searches enabled a 20 parts permillion mass tolerance for precursor ions. A carbamidomethylation of Cys was designated a fixed modification, whereas an acetylation at the protein N terminus and an oxidation of Met were designated as the variables. To further filter the data, the false discovery rate (FDR) and FDR accuracy were adjusted to 1% on the spectrogram. Peptides, protein levels, and an identified protein should contain at least one unique peptide. Pearson’s correlation and principal component analyses were performed to determine the replicates among the three samples for each group.

**Gene Ontology and Kyoto Encyclopedia of Genes and Genomes annotation Gand enrichment**

The Gene Ontology (GO) annotation proteome was derived from the UniProt-GOA database (<http://www.ebi.ac.uk/GOA/>). The IDs of the identified proteins were initially converted to UniProt IDs and then mapped to the corresponding GO IDs. For identified proteins that were not annotated in the UniProt-GOA database, InterProScan software (v5.33-72) was used to determine the GO function of the annotated protein using the protein sequence alignment method. The proteins were classified using GO annotation based on the following three categories: biological process (BP), cellular component (CC), and molecular function (MF). Statistical analysis and visualization were performed using the R4.2.1. clusterProfiler package and GOplot package v. 3.3.6.

**Domain annotation**

Protein domains were annotated using InterPro (http://www.ebi.ac.uk/interpro/) and the corresponding PfamScan tool.

**Subcellular localization**

To predict protein subcellular localization, we used WoLF PSORT (v3.0) (https://wolfpsort.hgc.jp/), which is an updated version of PSORT/PSORT II used in the prediction of eukaryotic sequences. Given that this analysis is specific to proto-karyon species, we used CELLO (http://cello.life.nctu.edu.tw/) subcellular localization prediction software.

**Protein–protein interaction networks**

For protein–protein interaction network analysis, a network interaction map was constructed and visualized using R software v. 3.6.3.

**qRT- PCR**

Total mRNA was extracted using TRIzol™ (15596018; Invitrogen, Waltham, MA, USA), following the manufacturer’s instructions. Equal amounts (1 μg) of mRNA were reverse transcribed to cDNA using an Evo M-MLV reverse transcription kit II (AG11711; Accurate Biology, Hunan, China). The resulting cDNA was used as a template for amplification using a Roche LightCycler 480 system (Hoffmann-La Roche, Basel, Switzerland) with a SYBR^®^ Green Premix Pro Taq HS qPCR Kit II (AG11702; Accurate Biology). The gene-specific primers used in this study are listed in Table I. All cycle threshold values of the analyzed genes were converted to delta-delta-cycle threshold values and normalized to GAPDH mRNA levels.

**Western blot analysis**

Cells were lysed using radioimmunoprecipitation assay lysis buffer (P0013C; Beyotime) supplemented with protease and phosphatase inhibitors (Roche Applied Science, Basel, Switzerland). Protein concentrations were determined using the BCA assay (23225; Thermo Fisher Scientific, Waltham, MA, USA). Subsequently, equal amounts of denatured protein were loaded onto 4–20% SmartPAGE™ Precast Protein Gel Plus (SLE020; Smart-Lifesciences, London, UK) and transferred to polyvinylidene difluoride membranes (1620177; Bio-Rad, Hercules, CA, USA). After blocking with 5% skimmed milk for 1 h, the membranes were incubated overnight at 4°C with the following primary antibodies: PGGT1 (PA5-56529, 1:500; Invitrogen), P-p44/42 mitogen-activated protein kinase (MAPK) (Erk1/2) (4370S, 1:1000; Cell Signaling Technology, Danvers, MA, USA), phospho-SAPK/c-Jun N-terminal kinase (JNK) (4668S, 1:1000; Cell Signaling Technology), P-p38 MAPK (4511S, 1:1000; Cell Signaling Technology), phospho-IκBα (2859, 1:1000; Cell Signaling Technology), IκBα (4814, 1:1000; Cell Signaling Technology), P-NF-κB p65 (3033S, 1:1000; Cell Signaling Technology), NF-κB p65 (8242, 1:1000; Cell Signaling Technology), and GAPDH (5174, 1:1000; Cell Signaling Technology). After washing, membranes were incubated with anti-rabbit IgG (7074, 1:2000; Cell Signaling Technology) and anti-mouse IgG (7076, 1:2000; Cell Signaling Technology) secondary antibodies. Protein bands were visualized using Clarity Max Western ECL Substrate (1705060; Bio-Rad). Then, using ImageJ software, the cumulative optical density (IOD) of each stripe will be automatically measured, that is, the sum of the optical densities of all pixels. In order to reduce the experimental error, the IOD value of the target protein was corrected by the IOD value of the reference protein GAPDH.

**Supplementary Data:**


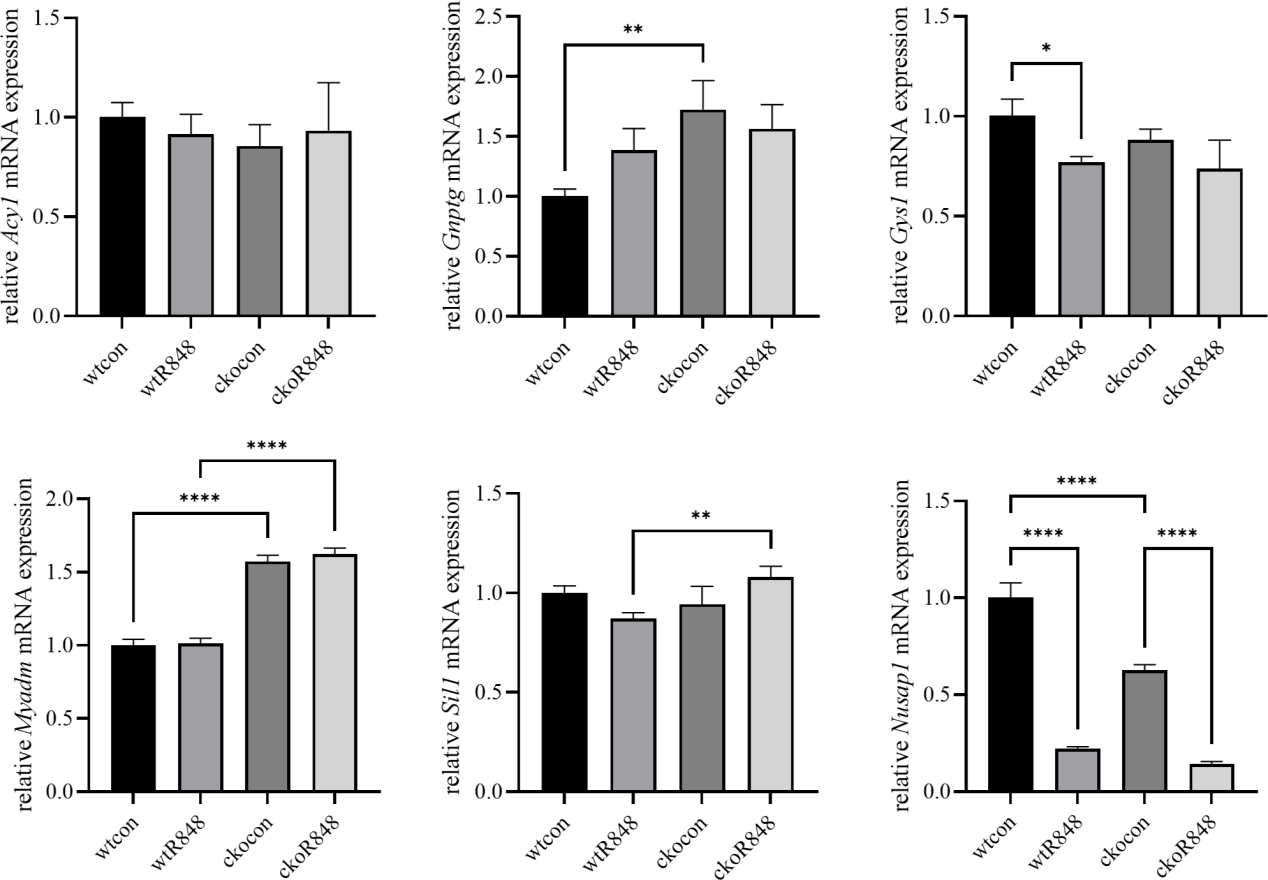


**Fig**. **S1**. Quantitative real-time PCR analysis of mRNA candidate proteins normalized to the expression of GAPDH mRNA levels. (*, *P* < 0.05; **, *P* < 0.01; ****, *P* < 0.0001)


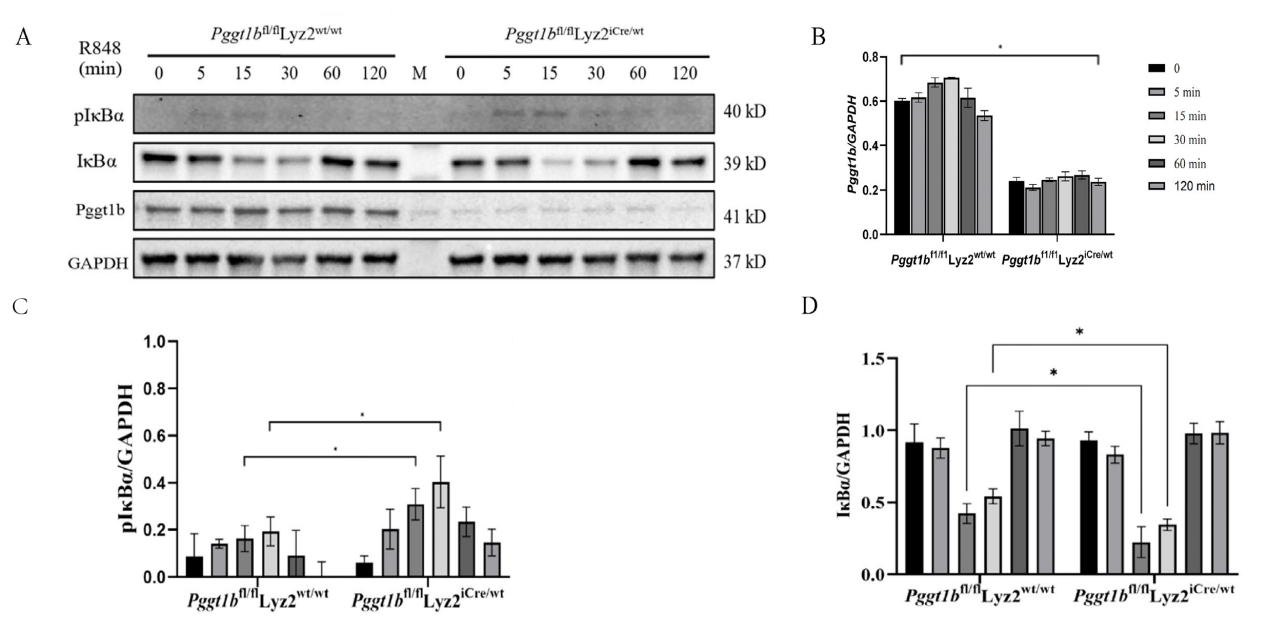


**Fig**. **S2**. NF-κB pathway is enhanced in Pggt1b-deficient BMDMs. (A) Western blot analysis of phosphorylated IκBα, IκBα, and Pggt1b in BMDM lysates stimulated (above lanes) with R848 (1 μg/mL). (B)-(D) Statistical analysis of interested protein levels was shown. GAPDH served as a loading control.(*, *P* < 0.05; **, *P* < 0.01; ***, *P* < 0.001; ****, *P* < 0.0001). The data represent the mean ± SD of three independent experiments (N=3, n=1), where each repetition involved the use of BMDM from one mouse per group.
